# Vocalization behaviors in captive bottlenose dolphins (*Tursiops truncatus*) and rough-toothed dolphins (*Steno bredanensis*)

**DOI:** 10.1371/journal.pone.0336138

**Published:** 2025-10-31

**Authors:** Weijie Fu, Zhongchang Song, Wenjie Xiang, Chuang Zhang, Wuyi Yang, Xiaohui Xu, Yu Zhang

**Affiliations:** 1 Key Laboratory of Underwater Acoustic Communication and Marine Information Technology of the Ministry of Education, College of Ocean and Earth Sciences, Xiamen University, Xiamen, China; 2 State Key Laboratory of Marine Environmental Science, College of Ocean and Earth Sciences, Xiamen University, Xiamen, China; 3 Shenzhen Research Institute of Xiamen University, Shenzhen, China; University of Naples Federico II: Universita degli Studi di Napoli Federico II, ITALY

## Abstract

Odontocetes rely on vocalizations for navigation, foraging, and communication. Their vocalization patterns are associated with environmental conditions and behavioral contexts, particularly in captive populations. This study investigated the vocalization behaviors of captive bottlenose dolphins (*Tursiops truncatus*) and rough-toothed dolphins (*Steno bredanensis*) using continuous acoustic monitoring. The focus was on their responses to human-involved training and feeding activities. Comparative analyses revealed that rough-toothed dolphins produced significantly more clicks and fewer whistles than bottlenose dolphins (*p* < 0.05). During training, bottlenose dolphins reduced their click rate by 41% but increased whistle production by 125%. In contrast, rough-toothed dolphins showed no significant change in click emissions (from 643.9/min to 597.4/min, *p* > 0.05), but significantly reduced whistles by 56% (*p* < 0.05). Neither species exhibited significant changes in vocalization during feeding. However, rough-toothed dolphins shifted their predominant whistle type from “constant” to “sinusoidal”, while bottlenose dolphins changed from “constant” to “upsweep” during feeding. These findings offer valuable insights expanding current knowledge of dolphin vocal patterns under captivity and establish baseline information potentially supporting acoustic assessment of captive dolphin welfare, particularly for the understudied rough-toothed dolphin.

## Introduction

Odontocetes evolved the ability to use sound for foraging, navigation, and communication [1]. They emit highly directional echolocation clicks while navigating and echolocating prey or targets of interest. Echolocation clicks are characterized by high-frequency, short-duration, and form trains [2]. This type of emission is often recognized as an indicator of odontocetes’ presence in passive acoustic monitoring, due to their high frequency of occurrence during echolocation. Most odontocete species, except for porpoises, produce continuous, narrow-band, and frequency-modulated whistles as communication signals [1]. Whistles can be readily identified and characterized by their distinctive frequency contours and are commonly categorized into six tonal types: constant, upsweep, downsweep, concave, convex, and sinusoidal [3–5]. Whistles have garnered significant research interest due to their important social functions in biological activities, such as collaborative foraging, reproductive gatherings, and mother-calf interactions [6–9].

Odontocetes exhibit adaptive modifications in their vocalization patterns in response to changing environmental conditions. For instance, beluga whales (*Delphinapterus leucas*) in extremely shallow waters (<1 m depth) produce significantly higher click rates (median ICI = 25.2 ms) compared to those in open waters (97.4 ms) [10,11]. Captive species such as the Baiji (*Lipotes vexillifer*), finless porpoises (*Neophocaena phocaenoides*), and bottlenose dolphins (*Tursiops truncatus)* have also been observed to exhibit similar acoustic adjustments, likely as an adaptation to the constrained echolocation ranges within artificial tank environments [12]. Furthermore, odontocete vocal behavior is influenced by ambient noise levels. Killer whales (*Orcinus orca*) and melon-headed whales (*Peponocephala electra*) have been reported to increase their vocal amplitude in response to elevated background noise [13–15].

Adjustments in odontocetes’ vocalizations are also associated with behavioral contexts [16]. Sperm whales (*Physeter macrocephalus*) dynamically adjust click intervals and acoustic properties during prey approach and capture phases [17]. False killer whales (*Pseudorca crassidens*), Killer whales, and bottlenose dolphins were reported to increase signal repetition rates and reduce output energy before prey interception [18,19]. Compared to free-ranging animals, captive odontocetes modify vocalizations primarily during interactions with humans, particularly in feeding and training sessions. For instance, captive Yangtze finless porpoises produced more clicks when socially separated during training/feeding sessions [20]. Increased vocalization rates and sound types were reported in captive beluga whales and Pacific white-sided dolphins (*Lagenorhynchus acutus*) during training and feeding sessions [21,22].

In summary, the vocal behavior of odontocetes is influenced substantially by environmental conditions and behavioral contexts. Monitoring their vocalization patterns can thus serve as a non-invasive auxiliary tool offering critical insights into their behavioral status and welfare [20,23–26]. Compared to the wild populations, captive odontocetes experience intensified interactions with humans, emphasizing the importance of assessing how captivity and associated human activities influence their acoustic behaviors.

In this study, continuous passive acoustic monitoring was conducted to investigate the vocalization patterns of captive bottlenose dolphins and rough-toothed dolphins (*Steno bredanensis*), with a particular focus on variations in vocalization rate and whistle type during feeding and training sessions. Bottlenose dolphins, among the most widely distributed odontocetes globally, are the most commonly maintained species in captivity. In contrast, rough-toothed dolphins are relatively understudied, and current knowledge of their vocal behavior is largely derived from studies on wild populations [4,16,27–32]. Information on the vocalizations of captive rough-toothed dolphins remains scarce. The present study provides essential baseline data that address current knowledge gaps in vocalization behaviors of captive dolphins, while also supporting the potential use of acoustic monitoring to assess animal welfare through identifying vocal abnormalities that may indicate welfare concerns. Furthermore, comparisons between these two distinct dolphin species provide insights into the potential interspecific differences or similarities in vocal modulation under identical human-care scenarios, which may help inform the development of species-specific management strategies.

## Materials and methods

### Facility and captivity management

**Fig 1:**
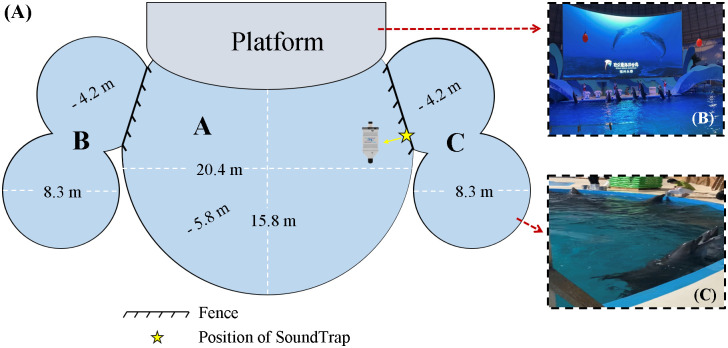
Dolphin enclosure facilities at Ocean World - Europark. (A) The dolphin pool at Ocean World – Europark; Field pictures of (B) training activities, and (C) feeding activities.

This study was conducted at Ocean World – Europark, Fuzhou, China. Dolphins were housed in a facility separated into three distinct regions by mesh fences interconnected through water channels (Fig 1A). Pool A is approximately 20.4 meters in length and 15.8 meters in width. Its upper side connects to the training platform, with the water depth maintained at 5.8 meters. Pools B and C are formed by two identical circular pools, each with a diameter of 8.3 meters. They are symmetrically positioned on either side of Pool A, with a water depth of approximately 4.2 meters.

Dolphins were primarily housed in Pool A and transferred to Pool C during feeding sessions. Pool B remained closed throughout the experimental period. Dolphins were generally fed 3–4 kg of thawed fish per day. The dolphin feeding amounts are adjusted based on age, gender, weight, season, and reproductive status for a healthy weight and optimal body condition. Daily food intake is accurately recorded and serves as a vital metric for health assessment. Basic health inspections, including visual checks of the skin, eyes, and blowhole, are performed routinely. Environmental enrichment is provided through the provision of various toys, including balls, floating rafts, and water curtains. Pool water quality is routinely monitored, with divers cleaning the pool walls approximately once per month.

### Data collection

In 2022, eight bottlenose dolphins were initially housed at the dolphin pool before being relocated to another oceanarium. Subsequently, eight rough-toothed dolphins replaced the bottlenose dolphins in the same facility. Acoustic recordings of these two dolphin groups was carried out at different times (Table 1): (1) bottlenose dolphins were recorded from 15:40 on 11 November 2021 to 08:00 on 13 November 2021, and (2) rough-toothed dolphins from 17:00 on 12 October 2022 to 19:00 on 15 October 2022. An omnidirectional underwater acoustic recorder, SoundTrap 300HF (Ocean Instruments Ltd., Whangateau, New Zealand), was used for acoustic data collection. The recorder has a linear frequency response from 20 Hz to 150kHz, with a sensitivity of −188 dB re 1 V/ μPa. The sampling rate was set at 576 kS/s, allowing continuous recording for approximately four days per full charge. The SoundTrap recorder was securely attached with waterproof tape at a depth of 2 m underwater, positioned at the mesh fence separating Pools A and C (Fig 1A). To minimize potential equipment damage from animal interaction, the recorder was placed on the opposite side of the fence from where the dolphins were kept.

**Table 1:** Timing of the recorded feeding and training events.

Monitored animals	Group size	Recording period	Feeding events				Training events			
			No.	Start time	End time	Duration/min	No.	Start time	End time	Duration/min
Bottlenose dolphins	8	2021/11/11 15:40 – 2021/11/13 08:00	1	2021/11/12 08:43	2021/11/12 09:25	42	1	2021/11/12 12:00	2021/11/12 12:10	10
Rough-toothed dolphins	8	2022/10/12 17:00 – 2022/10/15 19:00	1	2022/10/13 08:30	2022/10/13 09:00	30	1	2022/10/13 11:40	2022/10/13 11:54	14
							2	2022/10/13 15:53	2022/10/13 16:05	12
			2	2022/10/14 08:30	2022/10/14 09:00	30	3	2022/10/14 11:40	2022/10/14 11:54	14
							4	2022/10/14 15:50	2022/10/14 16:02	12
			3	2022/10/15 08:30	2022/10/15 09:00	30	5	2022/10/15 11:40	2022/10/15 11:52	12
							6	2022/10/15 15:52	2022/10/15 16:04	12

Throughout acoustic monitoring, the training and feeding events, as well as their precise start and end times, were accurately documented (Table 1). Training sessions typically last about 10–15 minutes, during which dolphins perform a series of trained behaviors in response to auditory and visual cues (e.g., whistles and hand signals) provided by the trainers. These behaviors included: I. Dolphins surfacing from underwater and maintaining a vertical upright posture while moving from the platform to the opposite side of the pool, then swimming back to the starting point; II. Dolphins synchronously leaping out of the water from underwater, flipping in midair, and then diving back in; III. Dolphins diving headfirst into the water and maintaining an inverted posture with their tails exposed above the surface while executing tail-wagging motions. Dolphins that successfully performed these behaviors received fish rewards as positive reinforcement (Fig 1B). The training process did not include any intentional or potential dolphin vocalization induction. In feeding events, dolphins were guided by trainers from Pool A into Pool C, where they were provided with freshly deceased fish (Fig 1C). Feeding sessions generally lasted from 30 to 42 minutes. Over the entire monitoring period, one training event and one feeding event were recorded for bottlenose dolphins, and a total of three training events and six feeding events were recorded for rough-toothed dolphins (Table 1). To facilitate further comparative analyses, two distinct temporal phases were defined for each recorded training and feeding event: (1) the 30-minute period before training and feeding (referred to as phase BE) and (2) the duration of the training and feeding activities (referred to as phase PE). In all recorded events, dolphins were free-swimming during phase BE.

### Data analyses

#### Acoustic data processing.

**Fig 2:**
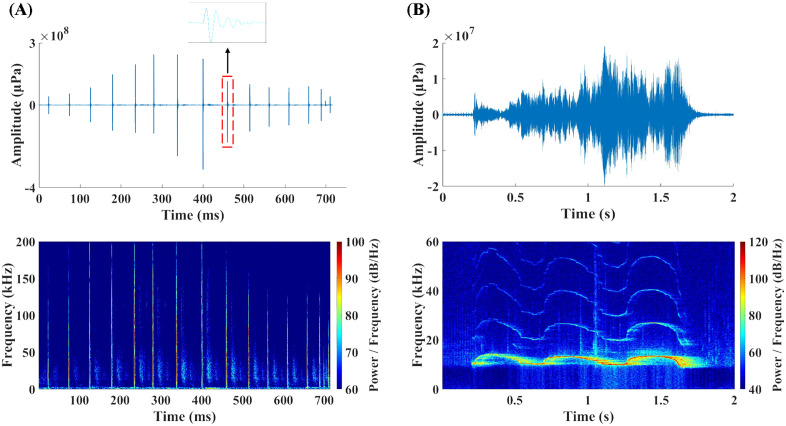
Representative vocalizations of dolphins: echolocation clicks and communication whistles. (A) A click train recorded during the acoustic monitoring and its spectrogram in the time-frequency domain; (B) A recorded whistle signal and its spectrogram in the time-frequency domain.

To mitigate the computational burden associated with processing large datasets, the original 30-minute WAV audio files from the SoundTrap recorder were divided into 10-minute segments prior to analysis. To obtain high-quality echolocation clicks, a Butterworth band-pass filter with a cut-off frequency of 2 kHz was applied to eliminate low-frequency noise. Additionally, given the frequency limitations of the hydrophone, frequency components above 150 kHz were also filtered out of the acoustic data. A custom MATLAB (MathWorks, Cambridge, MA) script was developed to extract echolocation clicks with a signal-to-noise ratio (SNR) exceeding 6 dB from each filtered data segment (Fig 2A). Detections of whistles were manually conducted in the time-frequency domain. The acoustic files were displayed in a 4-second time window to visualize the frequency spectrum using Adobe Audition software (Version 2021). A trained and experienced operator then visually scanned the audio spectrogram for the presence of whistles. Following previous studies, a continuous tonal contour without temporal breakpoints on the spectrogram was identified as a single whistle. Additionally, consecutive contours were also considered a single whistle if the gap between them was shorter than 200 ms and less than the duration of the contours. (Fig 2B) [3,5,25].

#### Sound data statistical analysis.

Click counts were determined in each 10-minute data segment throughout the monitoring period, and clicking rates (number of clicks per minute) were calculated. To evaluate variations in sound production associated with human-dolphin interactions, data segments corresponding specifically to phase BE (30-minute period before the event) and PE (duration of the event) of training/feeding events were picked out, according to recorded start and end times (Table 1). Clicking rates for phases BE and PE were computed, respectively.

**Fig 3:**
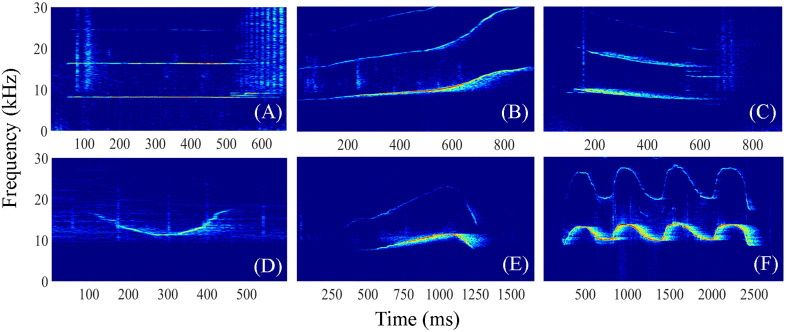
Spectrogram representation of the six whistle types. **(A)** constant, **(B)** upsweep, **(C)** downsweep, **(D)** concave, **(E)** convex, and **(F)** sinusoidal.

Whistle rates (whistles per minute) were quantified through visual inspection of spectrograms, both across the entire monitoring period and specifically for data segments representing phases BE and PE of training and feeding events. To investigate changes in whistle repertoires during interactions with humans, whistle contours were further manually categorized into six tonal types previously determined by Bazúa-Durán & Au [3–5] (Fig 3). The defined whistle types included: “Constant”- whistles with a maximum frequency change of no more than 1 kHz; “Upsweep”- whistles with a predominantly upward trend in frequency; “Downsweep”- whistles with a predominantly downward trend in frequency; “Concave”- whistles contour that has an initially mainly downward trend and then shifts to an upward trend; “Convex”- whistles contour that has an initially mainly upward trend and then shifts to a downward trend; and “Sinusoidal”- whistles contour with a shape similar to a sine signal, where the trajectory rises and then falls, or vice versa, with at least two inflection points. To ensure the reliability of classifications, two experienced operators familiar with dolphin whistles independently conducted whistle classification based on the same criteria, and the few cases with consistent in classifications were resolved through discussion between the two analysts and a third researcher. The final consensus classification was used for subsequent statistical analysis. The proportion of each whistle type, calculated as the number of whistles in each category relative to the total number of whistles, was determined for phases BE and PE during each training and feeding event.

Statistical analyses were conducted to examine variations in sound production rates and whistle-type distributions between phase BE and PE during training and feeding events. Additionally, differences in acoustic behaviour between dolphin species were evaluated. These statistical comparisons were performed using Independent Samples *t-*tests and Mann–Whitney *U* tests, with a significance level set at 0.05.

### Ethics statement

Research described in this study was reviewed and approved by the Fujian Provincial Department of Ocean and Fisheries, Fuzhou, China. Underwater acoustic monitoring carried out in this study was a completely passive and non-contact measurement method to record dolphin vocalizations with no negative effects on the dolphins, and no additional treatment was exerted on the dolphins during the entire monitoring period.

## Results

### Overall vocalizing rate

**Fig 4:**
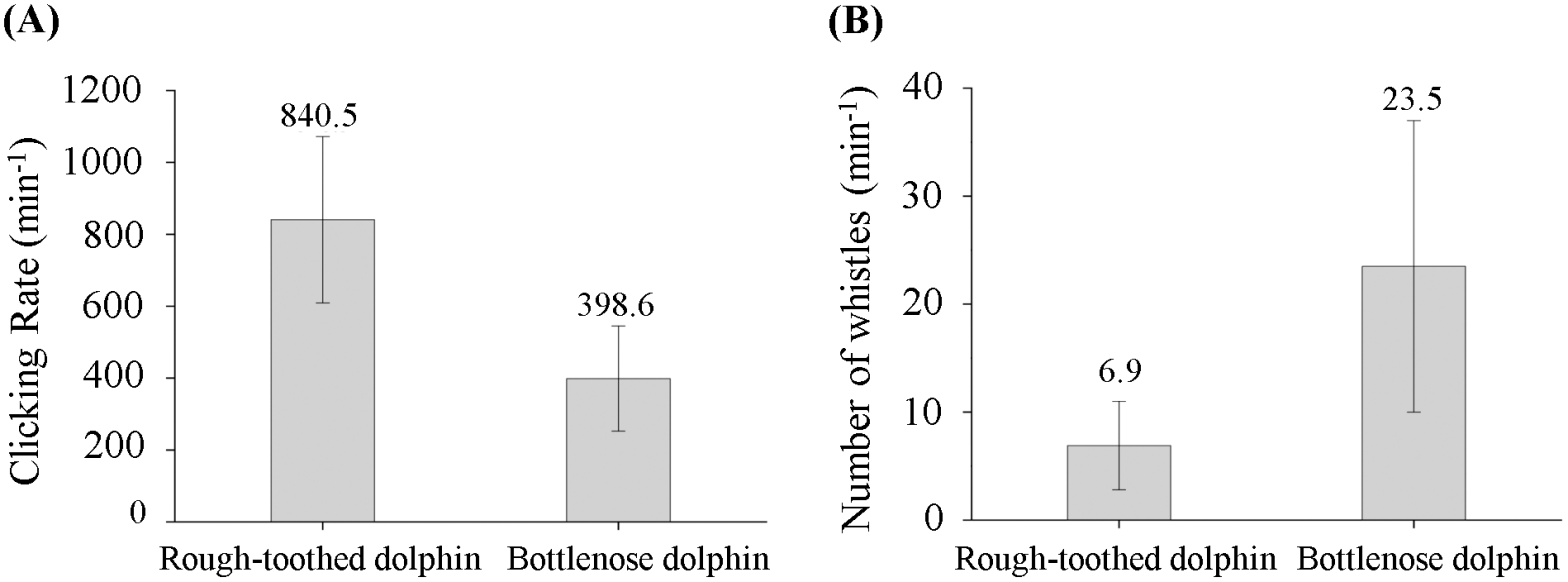
Underwater sound production rates of rough-toothed and bottlenose dolphins over the entire monitoring period. (A) The mean click emission rates of rough-toothed and bottlenose dolphins over the entire monitoring period. (B) The mean whistle emission rates of rough-toothed and bottlenose dolphins over the entire monitoring period.

Over the entire monitoring period, a total of 2300 min (230 segments) and 3930 min (393 segments) of acoustic data were analyzed for bottlenose and rough-toothed dolphins, respectively. Rough-toothed dolphins exhibited significantly higher acoustic activity in click production (840.5 ± 231.3 clicks/min) compared to bottlenose dolphins (398.6 ± 146.3 clicks/min), representing a 111% increase (Mann–Whitney U test; p < 0.05) (Fig 4A). Conversely, bottlenose dolphins produced whistles at a significantly higher rate of 23.5 ± 13.5/ min, compared to rough-toothed dolphins, which displayed a rate of 6.9 ± 4.1/ min over the entire monitoring period (241% more) (Mann–Whitney U test; p < 0.05) (Fig 4B).

### Vocalization in training events

#### Click emission rate.

**Fig 5:**
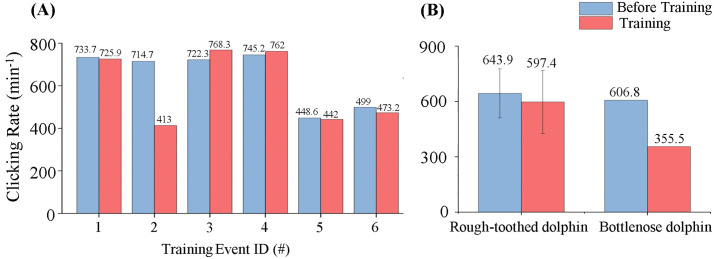
Click emission rates of rough-toothed and bottlenose dolphins in training events. (A) The click emission rates of rough-toothed dolphins before and during each training event. (B) The mean click emission rates of rough-toothed and bottlenose dolphins before and during training events.

During recorded training events, rough-toothed dolphins emitted clicks at a mean rate of 643.9 ± 133.1 clicks/min in phase BE (30 minutes prior to training) and 597.4 ± 171.1 clicks/min in phase PE (training duration) (Fig 5). Variations in the click production rate of the rough-toothed dolphin were not significant in the current research (Independent Samples t test; p > 0.05). In contrast, bottlenose dolphins exhibited a substantial decrease (41% reduction) in click emission rates from 606.8 clicks/min during phase BE to 355.5 clicks/min during phase PE (Fig 5B).

#### Whistle emission rate.

**Fig 6:**
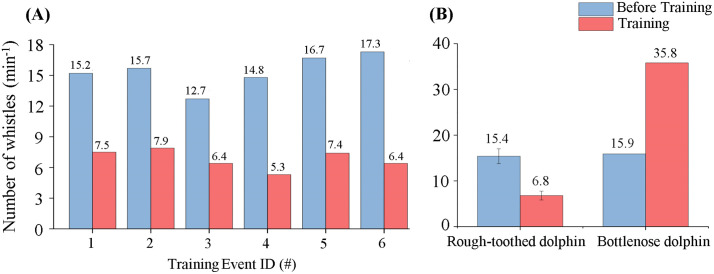
Whistle emission rates of rough-toothed and bottlenose dolphins in training events. (A) The whistle emission rates of rough-toothed dolphins before and during each training event. (B) The mean whistle emission rates of rough-toothed and bottlenose dolphins before and during training events.

Rough-toothed dolphins exhibited a significant decrease (56% reduction) in whistle rate from 15.4 ± 1.6 whistles/min in phase BE to 6.8 ± 1.0 whistles/min in phase PE (Independent Samples t test; p < 0.05). (Fig 6). Conversely, bottlenose dolphins increased whistle emissions substantially (125% increase) from 15.9 whistles/min during phase BE to 35.8 whistles/min during phase PE, a pattern clearly distinct from that observed in rough-toothed dolphins (Fig 6B).

#### Whistle type.

**Fig 7:**
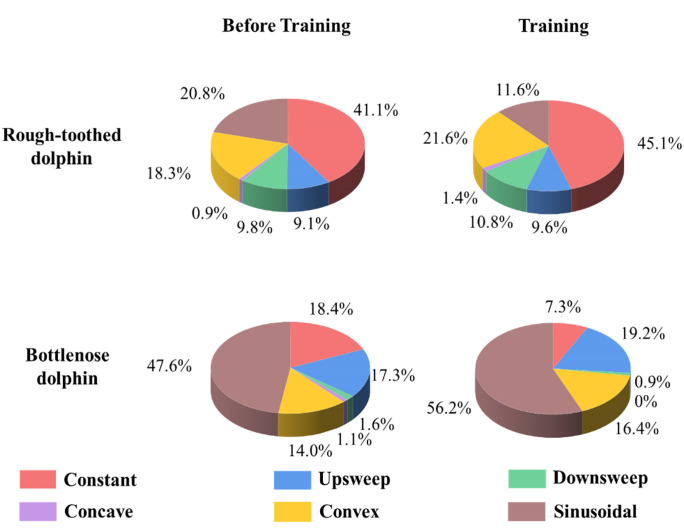
Distribution patterns of the whistle types of rough-toothed and bottlenose dolphins in training events. The numerical values around the pie chart denote the proportion of the corresponding whistle type.

In training events, no statistically significant differences were observed in the distribution patterns of the six whistle types for rough-toothed dolphins between phase BE (constant: 41.1 ± 11.2%; upsweep: 9.1 ± 0.9%; downsweep: 9.8 ± 2.8%; concave: 0.9 ± 0.7%; convex: 18.3 ± 3.4%; sinusoidal: 20.8 ± 8.7%) and phase PE (constant: 45.1 ± 7.8%; upsweep: 9.6 ± 2.1%; downsweep: 10.8 ± 1.7%; concave:1.4 ± 1.4%; convex: 21.6 ± 7.6%; sinusoidal: 11.6 ± 7.9%) (Independent Samples t test; p > 0.05) (Fig 7). Bottlenose dolphins represented a slight increase in sinusoidal whistle emissions (47.6% to 56.2%) and a reduction in constant whistles (18.4% to 7.3%) when training (Fig 7).

### Vocalization in feeding events

#### Click emission rate.

**Fig 8:**
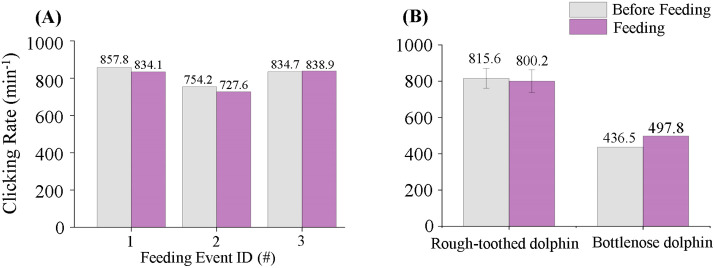
Click emission rates of rough-toothed and bottlenose dolphins in feeding events. (A) The click emission rates of rough-toothed dolphins before and during each feeding event. (B) The mean click emission rates of rough-toothed and bottlenose dolphins before and during feeding events.

No significant differences in click production rates were observed between pre-feeding (phase BE) and feeding (phase PE) periods for either dolphin species. Rough-toothed dolphins emitted clicks at similar rates before (815.6 ± 54.4 clicks/min) and during (800.2 ± 62.9 clicks/min) feeding events (Independent Samples t test; p > 0.05) (Fig 8). Similarly, bottlenose dolphins exhibited no significant change, producing clicks at rates of 436.5 clicks/min before feeding and 497.8 clicks/min during feeding (Fig 8B) (Independent Samples t test; p > 0.05).

#### Whistle emission rate.

**Fig 9:**
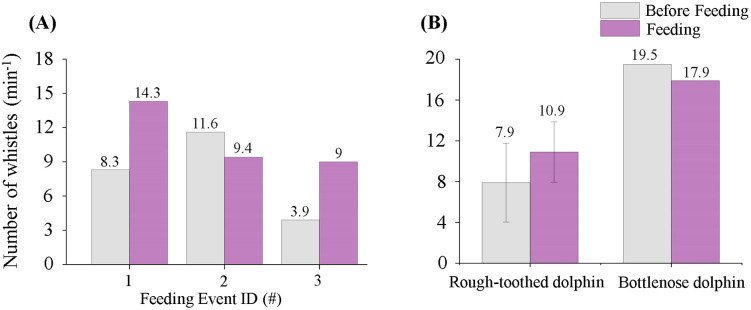
Whistle emission rates of rough-toothed and bottlenose dolphins in feeding events. (A) The whistle emission rates of rough-toothed dolphins before and during each feeding event. (B) The mean whistle emission rates of rough-toothed and bottlenose dolphins before and during feeding events.

Neither dolphin species demonstrated a statistically significant change in whistle emission rate in feeding events. Rough-toothed dolphins produced whistles at rates of 7.9 ± 3.9/ min during phase BE and 10.9 ± 3.0/ min during phase PE (Fig 9). Likewise, bottlenose dolphins produced whistles at rates of 19.5 whistles/min before feeding and 17.9 whistles/min during feeding, reflecting no significant difference (Independent Samples t test; p > 0.05) (Fig 9B).

#### Whistle type.

**Fig 10:**
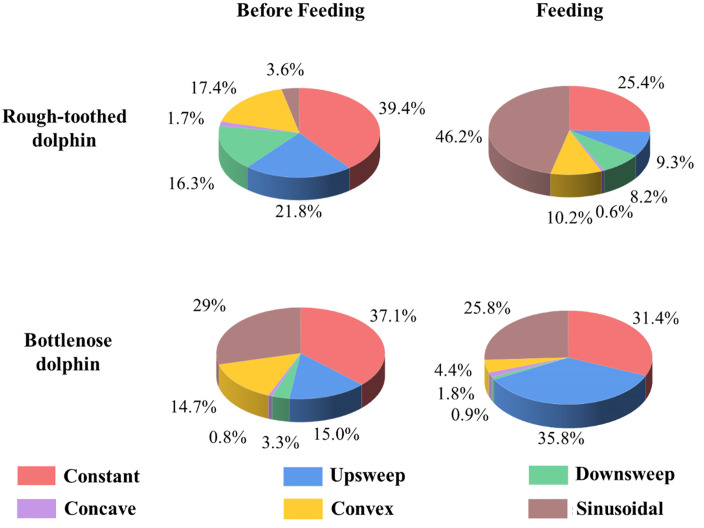
Distribution patterns of the whistle types of rough-toothed and bottlenose dolphins in feeding events. The numerical values around the pie chart denote the proportion of the corresponding whistle type.

In the recorded feeding events, rough-toothed dolphins predominantly emitted constant whistles during phase BE (39.4 ± 10.7%), followed by upsweep whistles (21.8 ± 3.4%). Downsweep (16.3 ± 10.4%) and convex (17.4 ± 1.1%) whistles occurred at comparable proportions, while sinusoidal (3.6 ± 3.4%) and concave whistles (1.7 ± 2.4%) were relatively uncommon. During feeding (phase PE), however, a significant shift was observed, with sinusoidal whistles increasing markedly to become the predominant whistle type (46.2 ± 14.4%) (Independent Samples t test; p < 0.05). In contrast, upsweep whistles significantly declined (9.3 ± 5.1%) (Independent Samples t test; p < 0.05). No statistically significant changes were found for the other whistle types (constant: 25.4 ± 13.4%; downsweep: 8.2 ± 0.6%; concave: 0.6 ± 0.6%; convex: 10.2 ± 4.4%) (Independent Samples t test; p > 0.05) (Fig 10).

For bottlenose dolphins, a substantial increase in upsweep whistles was observed in the recorded feeding sessions, with the proportion growing from 15.0% during phase BE to 35.8% during phase PE, resulting in a change in the most frequent whistle type from constant to upsweep. Conversely, decreases were observed in constant whistles (from 37.1% to 31.4%), downsweep (from 3.3% to 0.9%), convex (from 14.7% to 4.4%), and sinusoidal whistles (from 29.0% to 25.8%). Concave whistles exhibited a slight increase from 0.8% to 1.8% during feeding (Fig 10).

## Discussion

Increased echolocation click production associated with foraging activities has been extensively documented among various free-ranging odontocete species [15,33–37]. However, in this study, neither captive rough-toothed dolphins nor bottlenose dolphins exhibited significant differences in click emission rates before and during feeding sessions (Fig 8B). This discrepancy may arise from differences in foraging modes between captive and wild populations. Captive dolphins receive passive feeding from caregivers at scheduled times, reducing the necessity for echolocation-based prey detection, which likely results in a lower rate of click emissions compared to active foraging scenarios.

Limited previous research has addressed variations in the click production among captive dolphins during training or feeding sessions. Serres et al. (2021) reported that captive Yangtze finless porpoises produced substantially more clicks (a 312% increase) during training/feeding sessions compared to outside these sessions [20]. In contrast, rough-toothed and bottlenose dolphins in the present study did not exhibit a similar trend (Figs 5 and 8), indicating potential interspecies differences in acoustic responses to comparable contexts. Throughout the monitoring period, rough-toothed dolphins were notably more acoustically active in click emission than bottlenose dolphins (Fig 4A). Such differences may be attributed to interspecies variations, or possibly reflect the advanced cognitive capabilities and environmental detection efficiencies reported for bottlenose dolphins, which are known for their sophisticated behaviors and possess one of the largest brain index (brain weight/ (body size)^2/3^) among mammals (0.64), second only to humans (0.89) [38–44]. These cognitive advantages may enable bottlenose dolphins to achieve effective environmental perception with fewer echolocation clicks.

Whistle signals hold great significance for dolphins in group activities. Bottlenose dolphins are capable of coordinating actions in cooperative activities through whistle signals [45,46]. In our research, bottlenose dolphins produced far more whistle calls than rough-toothed dolphins over the entire monitoring period (Fig 4B). In addition, consistent with previous studies, bottlenose dolphins increased whistle emissions significantly during training sessions (Fig 6B) [25,47]. In contrast, rough-toothed dolphins significantly reduced whistle production during training (Fig 6B). This difference likely suggests greater inter-individual interactions and information exchanges among bottlenose dolphins during training, which may contribute to their higher training success rate compared to rough-toothed dolphins, as reported by trainers at Europark Marine Zoo.

The distribution patterns of whistle types, especially the characteristic (predominant) whistle type, were closely related to behavioral context. Previous research has shown that some cetaceans produce characteristic calls or increase call numbers during feeding to recruit conspecifics, signal feeding initiation, or manipulate prey [48–52]. Similarly, both dolphin species in our study shifted their predominant whistle types during feeding sessions (Fig 10). In compliance with earlier reports [16,24,53], bottlenose dolphins produced significantly more upsweep whistles during feeding sessions, with it became their predominant whistle type (Fig 10). Notably, this study reported, for the first time, a significant shift in rough-toothed dolphin whistle types during feeding, marked by a pronounced increase and dominance of sinusoidal whistles (Fig 10). Since the dolphins were fed by caretakers, rather than naturally hunting, these whistle changes likely serve a social function, broadcasting feeding events among the group. In rough-toothed dolphins, constant whistles remain the predominant whistle type outside of feeding sessions. This aligns with the observations of Miksis et al. (2002), which reported a higher prevalence of whistles with constant frequency components in captive dolphins and suggested that this may represent a learned behavior to mimic trainers’ whistles [54]. However, for our current study, it remains unclear whether the predominance of constant whistles represents an innate species-specific tendency or an imitation of the trainer’s whistle because the same phenomenon was also observed in wild rough-toothed dolphins [4]. While the current data does not allow for definitive confirmation of this hypothesis, it presents an insightful explanation that should be investigated in future studies.

It is important to acknowledge the limitations of the current study on the relatively short data collection period due to the COVID-19 prevention and control policies implemented at the Marine Zoo. While continuous acoustic monitoring captured multiple training and feeding events, providing a rich dataset suited to the focus of this study, dolphin vocal behavior may exhibit natural variation over extended periods. A longer sampling duration would be beneficial to more fully explore this potential variability, a direction that warrants attention in future research. The conclusions regarding bottlenose dolphins are derived from a single training and feeding event. Due to the lack of replication across multiple events, these findings should be interpreted as preliminary. Nevertheless, their strong consistency with previous research [16,24,53] provides robust corroboration for established knowledge of this species. Non-invasive underwater acoustic monitoring has proven effective in monitoring captive dolphin welfare [20,23–26]. The data presented here, particularly for the understudied rough-toothed dolphins, provide valuable baseline information critical for identifying acoustic abnormalities that may signal welfare concerns, while also offering important insights and a solid foundation for future hypothesis-driven research on cetacean vocal behavior in managed settings.

Future research with longer-term monitoring and a larger sample size of events is recommended to better account for the natural fluctuation in dolphin vocalizations and confirm the consistency and effect size of these vocalization changes, thus establishing more generalized baseline data to enhance the acoustic assessment framework for animal welfare evaluations. Moreover, controlled studies, such as examining vocalization patterns in individually isolated dolphins during training and feeding sessions, are needed to conclusively identify the drivers behind the acoustic changes observed in this study, as well as to elucidate their potential biological and social functions.

## Conclusions

The vocalization patterns of captive bottlenose dolphins and rough-toothed dolphins during human-involved training and feeding sessions were investigated in this study. The results revealed that rough-toothed dolphins exhibited significantly higher click rates but lower whistle rates compared to bottlenose dolphins. During training, bottlenose dolphins showed a marked decrease in click production alongside a substantial increase in whistle emission, whereas rough-toothed dolphins significantly reduced their whistle emissions. Furthermore, both species demonstrated notable shifts in whistle type distribution during feeding activities. Rough-toothed dolphins shifted their predominant whistle type from “constant” to “sinusoidal”, while bottlenose dolphins shifted from “constant” to “upsweep”. These findings contribute to a deeper understanding of the vocalization behaviors of captive dolphins and provide valuable baseline data potentially supporting the acoustic assessment of animal welfare, particularly for the understudied rough-toothed dolphin.

## Supporting information

S1 File Raw data of Fig 2. (XLSX)

S2 File Raw data of Fig 3A–3C. (XLSX)

S3 File Raw data of Fig 3D–3F. (XLSX)

S4 File Raw data of Figs 4–10. (XLSX)
